# Supplementary material for: Transcriptional Terminators Allow Leak-Free Chromosomal Integration of Genetic Constructs in Cyanobacteria
Source: Microorganisms. 2019 Aug 16;7(8):263. doi: 10.3390/microorganisms7080263 (PMC6723963; doi:10.3390/microorganisms7080263)
Supplement: Supplementary file 1 [file microorganisms-07-00263-s001.pdf]

Supplementary Materials:

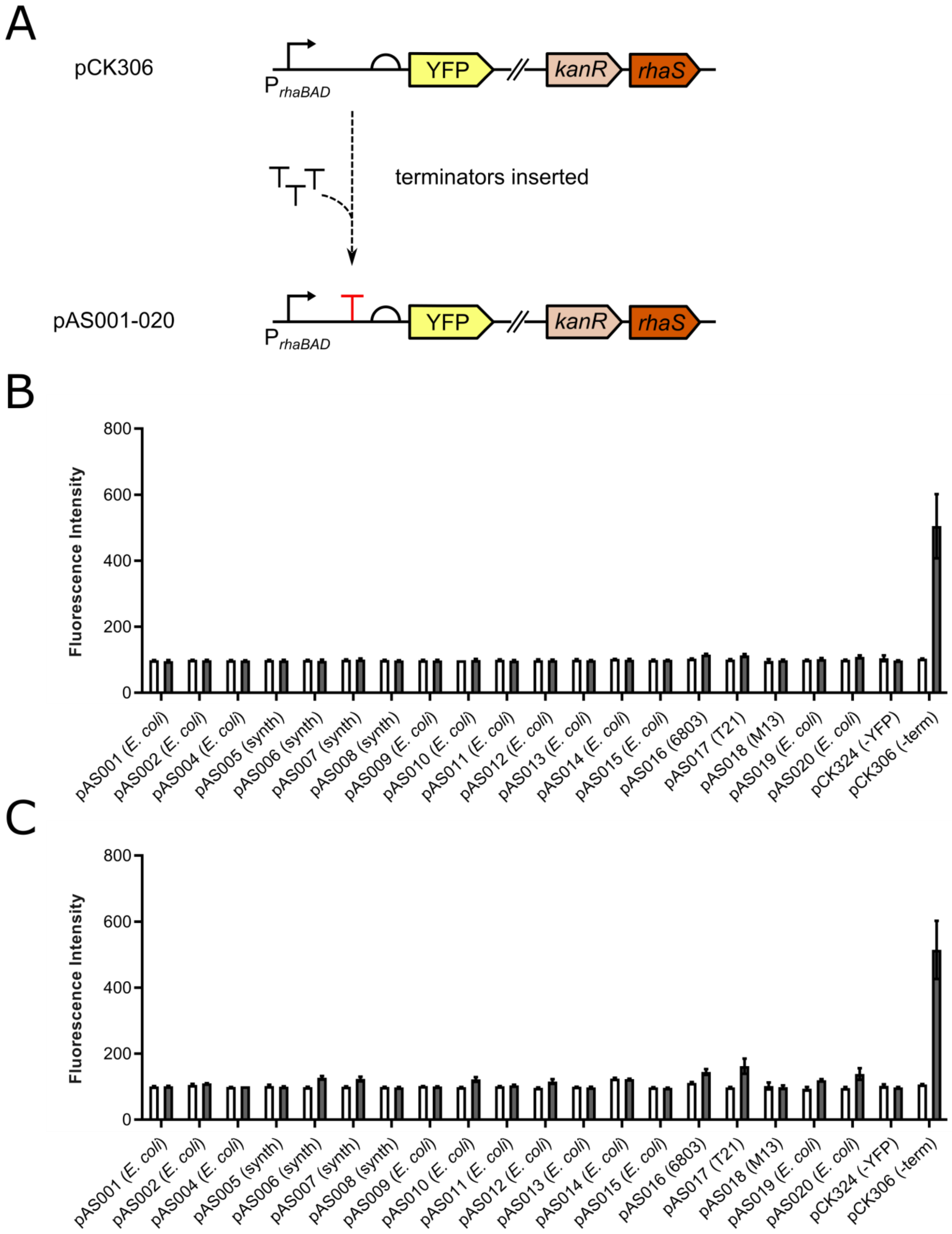

Figure S1. Efficiency of terminators in *E. coli* strains DH10B and MG1655. *E. coli* strains (A) DH10B and (B) MG1655 containing plasmids pAS001-002, pAS004-020 (terminator between *rhaBAD* promoter and RBS of YFP-encoding gene) were cultured in LB media supplemented with kanamycin and 0 mg/ml L-rhamnose (white bars) or 0.6 mg/ml L-rhamnose (black bars). Cells containing pCK324 (lacking *rhaBAD* promoter and therefore no YFP) and pCK306 (the parental vector with no terminator and therefore fully inducible with L-rhamnose) were used as controls. The fluorescence intensity of 10,000 cells (arbitrary units) from each culture was measured by flow cytometry after 6 h. Error bars shown are the standard deviation of the mean for three independent biological replicates. Key for SBOL glyphs used in figure: right-angled arrow represents a promoter;

T represents a terminator; semi-circle represents a ribosome-binding site (RBS); coloured blocks represent coding sequences or genes. Origin of each terminator in brackets after plasmid name: *E. coli*, synth (synthetic), 6803 (*Synechocystis* sp. PCC 6803), T21 (bacteriophage T21), M13 (bacteriophage M13).

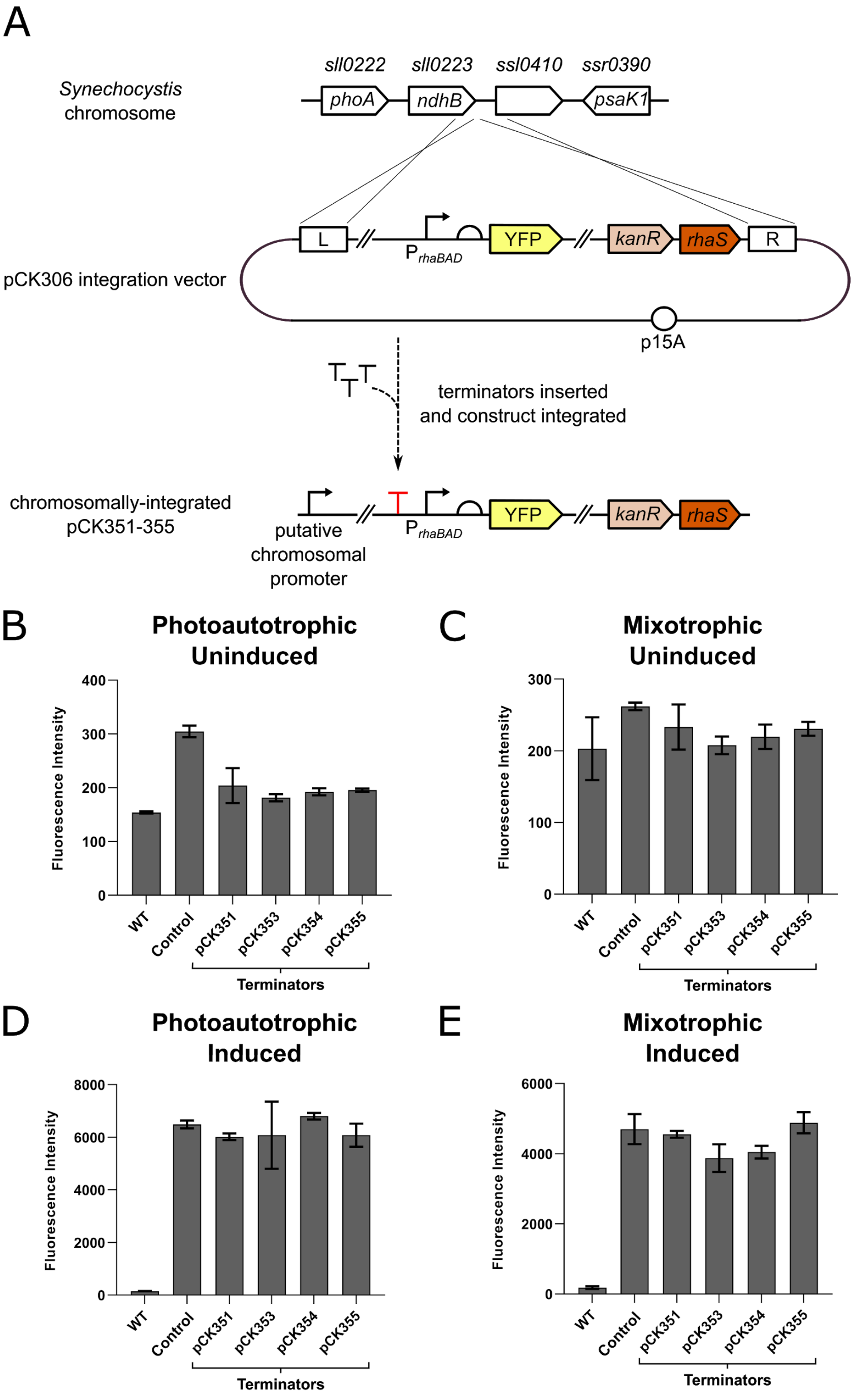
A
Synechocystis chromosome
sll0222
sll0223
ssl0410
ssr0390
phoA
ndhB
psaK1
pCK306 integration vector
L
PrhaBAD
YFP
kanR
rhaS
R
p15A
terminators inserted and construct integrated
chromosomally-integrated pCK351-355
putative chromosomal promoter
B
Photoautotrophic Uninduced
C
Mixotrophic Uninduced
D
Photoautotrophic Induced
E
Mixotrophic Induced
Fluorescence Intensity
WT
Control
pCK351
pCK353
pCK354
pCK355
Terminators

Figure S2. The effect of terminator insertion upstream of chromosomally-integrated DNA on transcriptional read-through from chromosomal promoters, at 192 h. (A) Detail showing the insertion of terminators into integration plasmid pCK306 upstream of the *rhaBAD* promoter. The resulting constructs pCK351, pCK353, pCK354 and pCK355 were integrated into the *Synechocystis* genome adjacent to the *ndhB* gene. (B) To test for transcriptional insulation from chromosomal promoters after integration, *Synechocystis* cells containing either pCK351, 353, 354 or 355 (each with one of four terminators inserted upstream of *rhaBAD* promoter) were cultured in BG11 media supplemented with kanamycin and no L-rhamnose, in photoautotrophic conditions and constant light. The fluorescence intensity (arbitrary units) of 10,000 cells measured after 192 h using flow cytometry and compared to wild-type and *Synechocystis* cells lacking YFP entirely and cells containing pCK306 (no terminator, *rhaBAD* promoter, YFP). (C) Equivalent experiment to (B) but strains cultured in BG11 supplemented with 5 mM D-glucose (mixotrophic growth). (D) The same strains of *Synechocystis* were cultured in BG11 media supplemented kanamycin and L-rhamnose to a final concentration of 0.6 mg/ml in photoautotrophic conditions and constant light. The fluorescence intensity (arbitrary units) of 10,000 cells measured after 192 h using flow cytometry and compared to wild-type and *Synechocystis* cells (lacking YFP entirely) and cells containing pCK306 (no terminator, *rhaBAD* promoter, YFP). (E) Equivalent experiment to (D) but strains cultured in BG11 supplemented with 5 mM D-glucose (mixotrophic growth). Error bars shown are the standard deviation of the mean for three independent biological replicates. Key for SBOL glyphs used in figure: right-angled arrow represents a promoter; T represents a terminator; semi-circle represents a ribosome-binding site (RBS); coloured blocks represent coding sequences or genes.

.

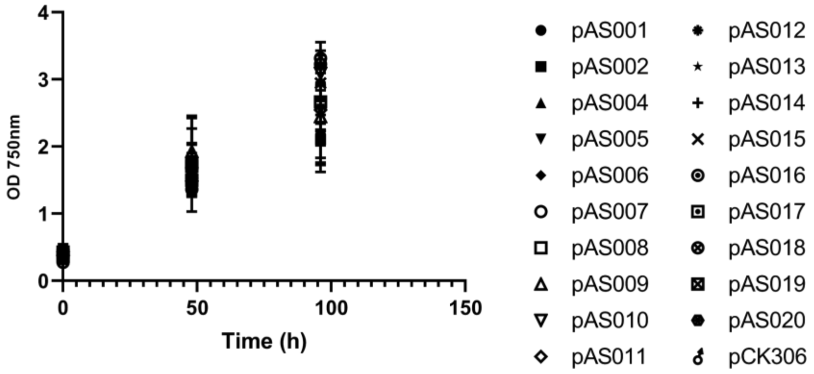

Figure S3. Growth of *Synechocystis* cells transformed with terminator plasmids, pAS001-pAS020. *Synechocystis* cells containing integrated terminator constructs from one of pAS001-002, pAS004-020 (terminator between *rhaBAD* promoter and RBS of YFP-encoding gene) or pCK306 (control, no terminator) were cultured in BG11 media supplemented with kanamycin and 0.6 mg/ml L-rhamnose in photoautotrophic conditions and constant light; and the optical density at 750 nm was monitored over time. Error bars represent the standard deviation of the mean for three independent biological replicates.

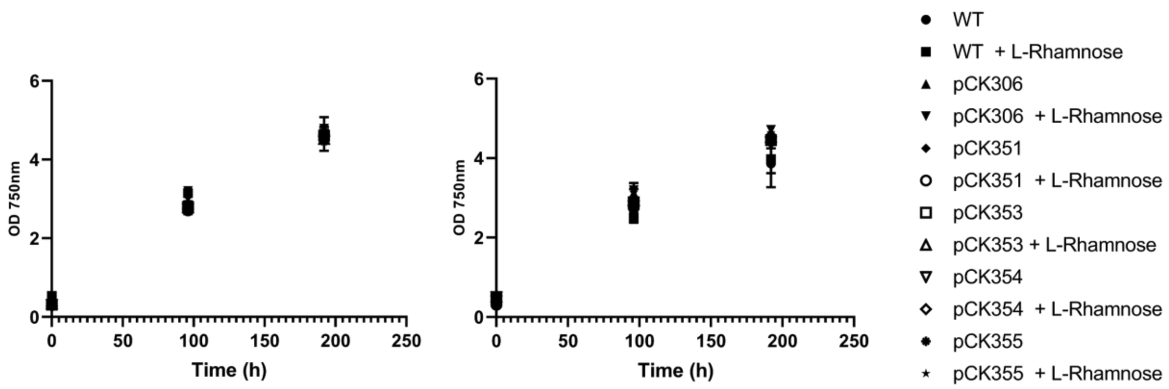

Figure S4. Growth of *Synechocystis* cells transformed with insulated plasmids pCK351, pCK353, pCK354 or pCK355. Wild-type (WT) *Synechocystis* cells, or cells containing integrated terminator constructs from one of pCK351, pCK353-355 plasmids (each with one of four Rho-independent terminators inserted upstream of the *rhaBAD* promoter) or pCK306 (control, no terminator) were cultured in BG11 media supplemented with kanamycin and 0 or 0.6 mg/ml L-rhamnose in photoautotrophic conditions and constant light; and the optical density at 750 nm was monitored over time. Error bars represent the standard deviation of the mean for three independent biological replicates.

Supplementary Materials and Methods

Plasmid Construction

A table of all plasmids and oligonucleotides (Table S1) is provided. Terminators were introduced as follows. Each terminator sequence was split in two at the hairpin-loop sequence and each part was included at the 5′ end of oligonucleotides that were then used to amplify pCK306. PCR fragments were then ligated by blunt-end ligation using the New England Biolabs site-directed mutagenesis kit and sequence verified.

Table S1. Plasmids and oligonucleotides used in this study.

Name	Details
pCK306	Medium copy plasmid (p15A), with 2054 nucleotides of homology to the *Synechocystis* sp. PCC 6803 chromosome, allowing integration of DNA after the first 34 nucleotides of ssl0410 (adjacent to *ndhB*), antibiotic resistance gene *kanR* encoding an aminoglycoside phosphotransferase, the *rhaBAD* promoter from *E. coli*, a synthetic RBS and eYFP, the *E. coli rhaS* RBS and gene inserted downstream of the *kanR* gene. [1]
pAS001-20	Detailed in Table 1
pCK351	As pCK306 but with terminator ECK120034435 inserted upstream of *rhaBAD* promoter
pCK353	As pCK306 but with terminator ECK120015170 inserted upstream of *rhaBAD* promoter
pCK354	As pCK306 but with terminator ECK120010799 inserted upstream of *rhaBAD* promoter
pCK355	As pCK306 but with the *ilvBN* terminator inserted upstream of *rhaBAD* promoter
oligoAS001	ACTGCAAGGTAGTGGACAAGACCGGCGGTCTTAAGTTTTTGGCTGAATACGACCAGTC TAAAAAG<br>Used in construction of pAS001
oligoAS002	AATGCGGTGGACAGGATCGGCGGTTTTCTTTTCTCTTCTCAAATGAATCGGGTAAGTTTA TAATATAC<br>Used in construction of pAS001
oligoAS003	AGGTGCGGGCTTTTTTCTGTGTTTCCTACGACCAGTCTAAAAAG<br>Used in construction of pAS002
oligoAS004	GACAGTGCGGGCTTTTTTTTTCGACCAAAGGATGAATCGGGTAAGTTTATAATATAC<br>Used in construction of pAS002
oligoAS007	TCTGGAATTTGGTACCGAGTACGACCAGTCTAAAAAG<br>Used in construction of pAS004
oligoAS008	AAAGAGACGCTGAAAAGCGTCTTTTTTCGTTTTGGTCCATGAATCGGGTAAGTTTATAAT ATAC<br>Used in construction of pAS004
oligoAS009	TCGGGAGGCCTCTTTTCTGGAATTTGGTACCGAGTACGACCAGTCTAAAAAG<br>Used in construction of pAS005
oligoAS010	AAGGGGGGCCTTTTTTCGTTTTGGTCCATGAATCGGGTAAGTTTATAATATAC<br>Used in construction of pAS005
oligoAS011	TCAGCGTCTTTTTTCGAAAATTTGGTACCGAGTACGACCAGTCTAAAAAG<br>Used in construction of pAS006

oligoAS012	AAAGCGTCTTTTTTCGTTTTGGTCCATGAATCGGGTAAGTTTATAATATAC<br>Used in construction of pAS006
oligoAS013	TCAGCGTCTTTTTTTTTTTTTTTGGTACCGAGTACGACCAGTCTAAAAAG<br>Used in construction of pAS007
oligoAS014	AAAGCGTCTTTTTTCGTTTTGGTCCATGAATCGGGTAAGTTTATAATATAC<br>Used in construction of pAS007
oligoAS015	TTTGGGAGGCCTTTTTTCGAAAATACGACCAGTCTAAAAAG<br>Used in construction of pAS008
oligoAS016	TCGGGGGGCCTTTTTTATTGATAACAAAAATGAATCGGGTAAGTTTATAATATAC<br>Used in construction of pAS008
oligoAS017	TCGACTGAGCCTTTCGTTTTATTTGATGCCTGGTACGACCAGTCTAAAAAG<br>Used in construction of pAS009
oligoAS018	AAGACTGGGCCTTTCGTTTTATCTGTTGTTTGTCGGTGAACGCTCTCTACTAGAGTCACAC<br>TGGCTCACCTTCGGGTGGGCCTTTCTGCGTTTATAATGAATCGGGTAAGTTTATAATATA<br>C<br>Used in construction of pAS009
oligoAS019	TCAGTCGCCTTAAAAATCAGTTACGACCAGTCTAAAAAG<br>Used in construction of pAS010
oligoAS020	TGAGTCGCCTTTTTTTTGTCTATGAATCGGGTAAGTTTATAATATAC<br>Used in construction of pAS010
oligoAS021	AGCGGCCTTTTTAGTTAGATCTACGACCAGTCTAAAAAG<br>Used in construction of pAS011
oligoAS022	CTGCGGCCTTTTTTCTTTTCACTATGAATCGGGTAAGTTTATAATATAC<br>Used in construction of pAS011
oligoAS023	AAGCGGGTTTTTTCGAAAATTGTTACGACCAGTCTAAAAAG<br>Used in construction of pAS012
oligoAS024	CGGCGGGTTTTTTTATAGCTAAAAATGAATCGGGTAAGTTTATAATATAC<br>Used in construction of pAS012
oligoAS025	ATCTTCCGGGGGCTTTCTCATGCGTTTACGACCAGTCTAAAAAG<br>Used in construction of pAS013
oligoAS026	CACCTTCCGGGGGCTTTTTTATTGCGCATGAATCGGGTAAGTTTATAATATAC<br>Used in construction of pAS013
oligoAS027	ACTCTGTCGCCTTTTTTCCTGACTCATAACTACGACCAGTCTAAAAAG<br>Used in construction of pAS014
oligoAS028	AATCTGTCGCCTTTTTTCTTTGCTTGCTTTATGAATCGGGTAAGTTTATAATATAC<br>Used in construction of pAS014
oligoAS029	TTCGACTGAGCCTTTCGTTTTATTTGATGCCTGGTACGACCAGTCTAAAAAG<br>Used in construction of pAS015
oligoAS030	AGACTGGGCCTTTCGTTTTATCTGTTGTTTGTCGGTGAACGCTCTCATGAATCGGGTAAGT<br>TTATAATATAC<br>Used in construction of pAS015
oligoAS031	AAACCTCCCTGGGATTTTATGAGAAAAGTGGTAGTCGGTCTACTAAACCTACGACCAGT

	CTAAAAAG<br>Used in construction of pAS016
oligoAS032	CGGCCTCCCTTTTTTTCACTTGCTAAGCTCTCTTTCGTTTATGAATCGGGTAAGTTTATAAT ATAC<br>Used in construction of pAS016
oligoAS033	AGAGTCACTAACGGCAGCTTATGCGAATAGTGTTGCTACTTGCTCAATTACGACCAGTCT AAAAAG<br>Used in construction of pAS017
oligoAS034	TAAGTTGCAACGGTGGCTTTTTTATATGAATCGGGTAAGTTTATAATATAC<br>Used in construction of pAS017
oligoAS035	AAGGAGCCTTTAATTGTATCGGTTTATCAGCTTGCTTTTACGACCAGTCTAAAAAG<br>Used in construction of pAS018
oligoAS036	TTGGAGCCTTTTTTTTTGGAGATTTTCAACATGAAAAAATTATTATTATGAATCGGGTAA GTTTATAATATAC<br>Used in construction of pAS018
oligoAS037	TCGGTGCGGGGGTCTTTACGACCAGTCTAAAAAG<br>Used in construction of pAS019
oligoAS038	AAGGTCCGGGGGTTTTTTTTATGAATCGGGTAAGTTTATAATATAC<br>Used in construction of pAS019
oligoAS039	AAGCGGGTTTTTACGTATACGACCAGTCTAAAAAG<br>Used in construction of pAS020
oligoAS040	CGGCGGGTTTTTACTTTATGAATCGGGTAAGTTTATAATATAC<br>Used in construction of pAS020

Supplementary References

1. Kelly, C.L.; Taylor, G.M.; Hitchcock, A.; Torres-Méndez, A.; Heap, J.T. A Rhamnose-Inducible System for Precise and Temporal Control of Gene Expression in Cyanobacteria. *ACS Synth. Biol.* **2018**, *7*, 1056–1066.
